# Plant Signals Anticipate the Induction of the Type III Secretion System in Pseudomonas syringae pv. *actinidiae*, Facilitating Efficient Temperature-Dependent Effector Translocation

**DOI:** 10.1128/spectrum.02073-22

**Published:** 2022-10-26

**Authors:** Maria Rita Puttilli, Davide Danzi, Cristiana Correia, Jessica Brandi, Daniela Cecconi, Marcello Manfredi, Emilio Marengo, Conceição Santos, Francesco Spinelli, Annalisa Polverari, Elodie Vandelle

**Affiliations:** a Department of Biotechnology, University of Verona, Verona, Italy; b Department of Biology, LAQV-REQUIMTE, Faculty of Sciences, University of Porto, Porto, Portugal; c Department of Agricultural Sciences, Alma Mater Studiorum University of Bologna, Bologna, Italy; d Department of Translational Medicine, Center for Translational Research on Autoimmune & Allergic Diseases (CAAD), University of Piemonte Orientale, Novara, Italy; e Department of Science and Technological Innovation, University of Piemonte Orientale, Alessandria, Italy; USDA—San Joaquin Valley Agricultural Sciences Center

**Keywords:** bacterial virulence, signaling, plant-pathogen interactions, plant-bacteria communication, environmental signals, virulence regulation, virulence timing

## Abstract

Disease resistance in plants depends on a molecular dialogue with microbes that involves many known chemical effectors, but the time course of the interaction and the influence of the environment are largely unknown. The outcome of host-pathogen interactions is thought to reflect the offensive and defensive capabilities of both players. When plants interact with Pseudomonas syringae, several well-characterized virulence factors contribute to early bacterial pathogenicity, including the type III secretion system (T3SS), which must be activated by signals from the plant and environment to allow the secretion of virulence effectors. The manner in which these signals regulate T3SS activity is still unclear. Here, we strengthen the paradigm of the plant-pathogen molecular dialogue by addressing overlooked details concerning the timing of interactions, specifically the role of plant signals and temperature on the regulation of bacterial virulence during the first few hours of the interaction. Whole-genome expression profiling after 1 h revealed that the perception of plant signals from kiwifruit or tomato extracts anticipated T3SS expression in P. syringae pv. *actinidiae* compared to apoplast-like conditions, facilitating more efficient effector transport *in planta*, as revealed by the induction of a temperature-dependent hypersensitive response in the nonhost plant Arabidopsis thaliana Columbia-0 (Col-0). Our results show that in the arms race between plants and bacteria, the temperature-dependent timing of bacterial virulence versus the induction of plant defenses is probably one of the fundamental parameters governing the outcome of the interaction.

**IMPORTANCE** Plant diseases—their occurrence and severity—result from the impact of three factors: the host, the pathogen, and the environmental conditions, interconnected in the disease triangle. Time was further included as a fourth factor accounting for plant disease, leading to a more realistic three-dimensional disease pyramid to represent the evolution of disease over time. However, this representation still considers time only as a parameter determining when and to what extent a disease will occur, at a scale from days to months. Here, we show that time is a factor regulating the arms race between plants and pathogens, at a scale from minutes to hours, and strictly depends on environmental factors. Thus, besides the arms possessed by pathogens and plants *per se*, the opportunity and the timing of arms mobilization make the difference in determining the outcome of an interaction and thus the occurrence of plant disease.

## INTRODUCTION

The course and outcome of host-pathogen interactions are thought to be determined mainly by the ability of each player to adapt existing or newly acquired genetic resources to provide enhanced offensive or defensive capabilities (1). The genes and effector molecules involved in such interactions have been investigated in the hope of developing new strategies for disease control in plants, but this line of inquiry has yielded few practical improvements. The comparative analysis of bacterial genomes has identified many genes involved in plant-microbe interactions, which provide insight into bacterial host range and virulence. However, further investigation is required to understand the dynamics of gene expression during interactions and the functions of effector proteins in both partners. In the interaction between a host plant and Pseudomonas syringae, several virulence factors contribute to early bacterial pathogenicity. For successful infection, P. syringae relies on a large repertoire of virulence effectors, which mainly act to suppress plant immunity and construct a favorable niche for bacterial growth within the apoplast (2). Such effectors are translocated into host cells via the type III secretion system (T3SS) (3), which requires an activating signal to achieve competence. Comparative microarray analysis based on publicly available genome data revealed that the *hrp*/*hrc* gene cluster (encoding the T3SS) is strongly induced in biovar 3 of the kiwifruit bacterial canker pathogen Pseudomonas syringae pv. *actinidiae* but only weakly induced in biovar 1 and unaffected in biovar 2, revealing differences in transcriptional responsiveness to apoplast-like conditions among the three biovars, particularly in the context of T3SS activation (4). Moreover, a reporter gene construct combining the promoter of the *hrpA1* gene (encoding one of the main components of the T3SS pilus) with the gene for green fluorescent protein (GFP) as an indicator of P. syringae pv. *actinidiae* virulence induction confirmed that one or more signals present in kiwifruit extracts boost *hrpA1* promoter activity in P. syringae pv. *actinidiae* biovar 3 (4). Although biovar 1 induces weaker T3SS-related gene expression in minimal medium than does biovar 3, it responds similarly to kiwifruit extracts, suggesting the presence of common sensors in the two biovars. In contrast, the *hrpA1* promoter remained inactive in biovar 2 cells under all conditions tested (4). P. syringae pv. *actinidiae* is therefore an ideal model for studies involving the induction of bacterial T3SS-related genes during interactions with plants, because the three biovars respond differently to minimal growth medium and/or kiwifruit extracts.

Although the T3SS activation process is poorly understood, there is compelling evidence that activation occurs following the perception of target cells (5, 6). Accordingly, the *hrp* gene cluster in P. syringae is controlled by plant signals and multiple physiological and environmental factors, specifically pH, osmotic potential, and catabolite repression. All *hrp*/*hrc* genes are induced *in vitro* under conditions that simulate the leaf apoplast environment, widely described as “minimal medium” (7, 8). Moreover, the *hrp* genes of the plant pathogen Ralstonia solanacearum are under the genetic control of *hrpB*, a regulatory gene whose expression is induced when bacteria are cocultivated with plant cell suspensions, due to the recognition of unidentified nondiffusible signals in the plant cell wall (9). Despite the evidence described above, transcriptomic analysis with bacteria grown under different conditions does not always show the induction of T3SS-related genes *in planta* or in the presence of plant extracts *in vitro* (10, 11). In some cases, the absence of *hrp* gene induction may result from the cultivation of bacteria in minimal medium, which already represents optimal conditions for the induction of T3SS-related genes. Alternatively, the inability to detect *hrp* gene induction *in planta* may indicate that induction is transient and subsides 48 to 72 h after inoculation. Indeed, large-scale transcriptome profiling experiments, though establishing a strong correlation between bacterial transcriptome profiles *in planta* at 6 h and further bacterial growth (12, 13), have mostly represented samples prepared several hours after infection or cultivated for several hours in the presence of plant extracts, thus obscuring the very early stages of the interaction.

Previous data obtained in P. syringae pv. *actinidiae* biovar 3 with the p*hrpA1*::gfp^c^ reporter system showed that the addition of kiwifruit extract to an apoplast-like medium led to a significant increase in fluorescence (and thus promoter activity) after 1 h, while this occurred later in a simple minimal medium (4). Thus, to address the lack of data covering the very early stages of plant-microbe interactions, we profiled the transcriptome of P. syringae pv. *actinidiae* biovar 3 cells grown in an apoplast-like medium, which revealed the induction of T3SS-related gene expression after only 1 h in the presence of kiwifruit or tomato leaf extract.

This finding suggests that the interaction with a host plant anticipates the T3SS-related gene expression already induced by apoplast-like conditions, as confirmed by an increase in secreted protein abundance. Moreover, given that phytopathogenic bacteria must overcome plant defenses that suppress T3SS activity, the induction of T3SS-related gene expression by plant extracts suggests that P. syringae pv. *actinidiae* enhances the activity of T3SS *in planta*, as revealed by the temperature-dependent hypersensitive response observed in the nonhost plant Arabidopsis thaliana Columbia-0 (Col-0).

## RESULTS

### Kiwifruit and tomato extracts enhance gene expression in P. syringae pv. *actinidiae* biovar 3 after only 1 h.

**FIG 1:**
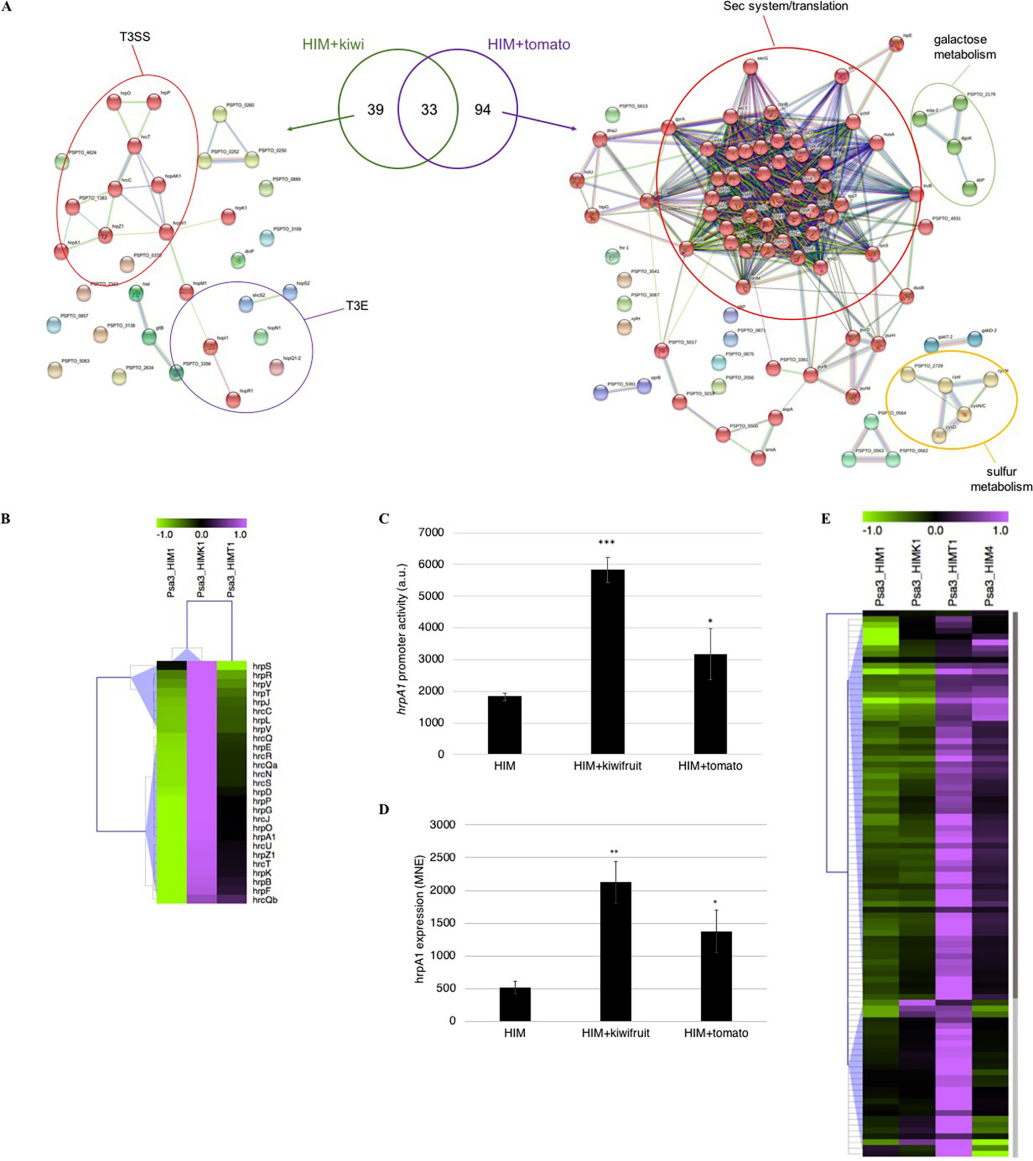
Kiwifruit and tomato extracts upregulate virulence-related genes after only 1 h in P. syringae pv. *actinidiae* (Psa) grown under apoplast-like conditions. (A) Common and specific genes upregulated in P. syringae pv. *actinidiae* biovar 3 by kiwifruit (left) and tomato (right) extracts and their functional relationship, analyzed using STRING. The main categories (T3SS, type III secretion system; T3E, type III effectors) are highlighted with colored circles. The Venn diagram, generated using Draw Venn Diagram, shows the common and unique upregulated genes for the two treatments. (B) Hierarchical clustering of the absolute expression levels of P. syringae pv. *actinidiae* biovar 3 *hrp*/*hrc* genes after incubation for 1 h in minimal (*hrp*-inducing) medium (HIM1) or HIM supplemented with kiwifruit (HIMK1) or tomato (HIMT1) extract. (C) Activity of the *hrpA1* promoter in a P. syringae pv. *actinidiae* biovar 3 strain carrying the p*hrpA1*::*gfp* reporter system after incubation for 1 h in HIM or HIM supplemented with kiwifruit or tomato extract. (D) Expression of *hrpA1* in P. syringae pv. *actinidiae* biovar 3 after incubation for 4 h in HIM or HIM supplemented with kiwifruit or tomato extract. The gene expression was analyzed by real-time qPCR using *rpoD* as a housekeeping gene for normalization and is conveyed as the mean normalized expression (MNE). (E) Hierarchical clustering of the absolute expression level of genes upregulated by tomato extract in P. syringae pv. *actinidiae* biovar 3 grown in HIM for 1 h (HIM1) or 4 h (HIM4) or in HIM1 supplemented with kiwifruit (HIMK1) or tomato (HIMT1) extract. In panels B and E, clusters were generated using MeV with normalized fluorescence values, and blue triangles indicate the different clusters. In panels C and D, the asterisks indicate statistically significant differences between the treated and untreated samples according to a Student’s *t* test (**, *P* < 0.05; *, *P* < 0.1).

The treatment of P. syringae pv. *actinidiae* biovar 3 cells with kiwifruit or tomato extracts led to the modulation of several genes after only 1 h compared with growth in minimal medium. We detected 73 differentially expressed genes (DEGs) (72 upregulated and 1 downregulated) in the presence of kiwifruit extract and 186 DEGs (127 upregulated and 59 downregulated) in the presence of tomato extract (see Data Sets S1 and S2 in the supplemental material). Among the upregulated genes, 33 were common to both treatments, whereas 39 were specifically induced by the kiwifruit extract and 94 by the tomato extract (Fig. 1A). The genes showing a statistically significant induction in response to the extracts were analyzed using the STRING database to identify clusters of associated proteins, revealing multiple functional clusters involved in a variety of pathophysiological processes. Among the genes induced by kiwifruit extract, the most obvious STRING cluster contained 25 proteins associated with the T3SS and type III effectors (Fig. 1A, left; red and purple circles, respectively). Among the genes induced by tomato extract, STRING analysis revealed proteins involved in translation and components of the Sec system, representing ~50% of the upregulated genes (Fig. 1A, right; red circle). The other two functionally related groups featured proteins associated with sulfur metabolism (Fig. 1A, right; yellow circle) and galactose metabolism (Fig. 1A, right; green circle). Finally, the 33 genes upregulated by both extracts included several encoding type III effectors and cognate chaperones, as well as the type III helper protein HopP1 (Data Set S3).

We focused on the *hrp*/*hrc* genes, the major category of P. syringae pv. *actinidiae* biovar 3 genes induced by kiwifruit extract. Although not every gene in the *hrp*/*hrc* cluster was differentially expressed, the transcripts were nevertheless always more abundant in the presence of plant extracts than in minimal medium (*hrp*-inducing medium HIM plus kiwi extract with 1-h incubation [HIMK1] versus HIM1 and HIM1 plus tomato extract [HIMT1] versus HIM1; Fig. 1B). The significance of this induction was confirmed by analyzing *hrpA1* promoter activity in a bacterial strain carrying the construct p*hrpA1*::GFP, as well as *hrpA1* expression using quantitative real-time PCR (qPCR). These experiments confirmed that *hrpA1* is upregulated in the presence of each extract, although to a different extent (Fig. 1C and D). The genes induced only by the tomato extract (Psa3_HIMT1) were generally upregulated more strongly than those induced only by the kiwifruit extract (Psa3_HIMK1), but the transcripts in the latter group were still more abundant than in cells incubated for 1 h in minimal medium (Psa3_HIM1) (Fig. 1D). Most of these transcripts also became more abundant when the incubation period was extended to 4 h (Psa3_HIM4). These results indicate that the extracts share common molecules that can induce bacterial signaling pathways, including the T3SS, but the quantity of the shared molecules varies. Interestingly, the genes upregulated by kiwifruit and tomato extracts were also induced in P. syringae pv. *tomato* DC3000 grown *in planta* for 6 h (12) (Fig. S1), suggesting that the genes play a functional role during the first phase of interaction, as expected for the T3SS, supporting the use of plant extracts as a model to study the influence of plant signals on bacterial behavior.

### Kiwifruit extract anticipates the P. syringae pv. *actinidiae* biovar 3 response and triggers faster induction of bacterial virulence.

**FIG 2:**
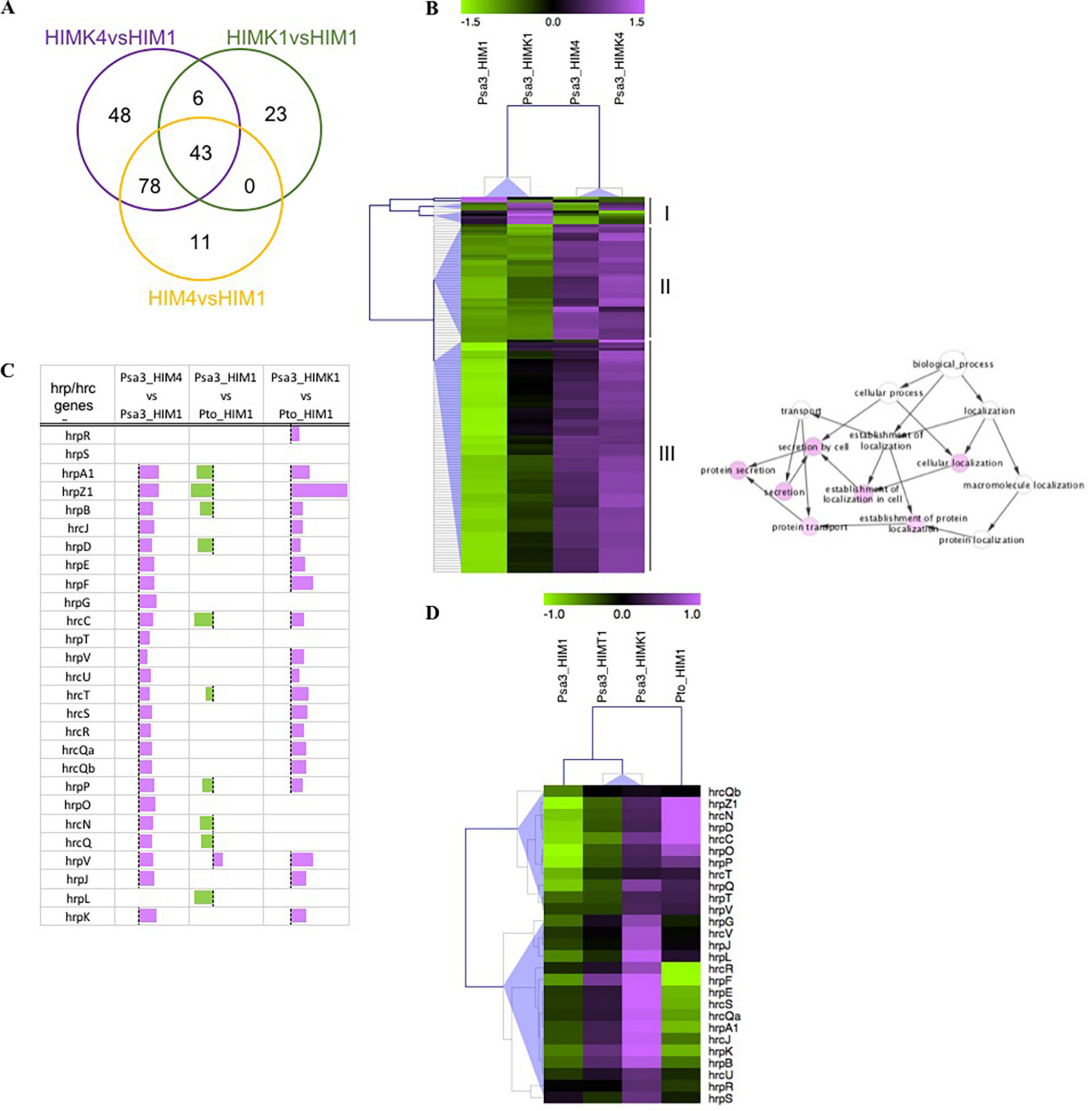
Kiwifruit extract anticipates T3SS-related gene expression and speeds up the induction of virulence in P. syringae pv. *actinidiae* (Psa) to match that in P. syringae pv. *tomato* DC3000 (Pto). (A) Common and specific genes upregulated in P. syringae pv. *actinidiae* biovar 3 incubated in minimal medium for 4 h (HIM4 versus HIM1) or in the presence of kiwifruit extract for 1 h (HIMK1 versus HIM1) or 4 h (HIMK4 versus HIM1). The Venn diagram was generated using Draw Venn Diagram. (B) Hierarchical clustering of the absolute expression level of P. syringae pv. *actinidiae* biovar 3 genes upregulated after 1 and 4 h in minimal (*hrp*-inducing) medium (HIM1 and HIM4) or HIM supplemented with kiwifruit extract (HIMK1 and HIMK4). Clusters were generated using MeV with normalized fluorescence values, and blue triangles indicate the different clusters. For each cluster (I to III), the functional category enrichment was analyzed using BINGO (right). (C) Graphical representation of the differential expression of *hrp*/*hrc* genes in P. syringae pv. *actinidiae* biovar 3 grown in minimal medium for 1 or 4 h (Psa3_HIM1 or Psa3_HIM4) or in the presence of kiwifruit extract for 1 h (Psa3_HIMK1) and in P. syringae pv. *tomato* DC3000 grown in minimal medium for 1 h (Pto_HIM1). Each bar represents the log_2_ fold change of expression for the corresponding gene. (D) Hierarchical clustering of the absolute expression level of *hrp/hrc* genes in P. syringae pv. *actinidiae* biovar 3 and P. syringae pv. *tomato* DC3000 grown in minimal medium (Psa3_HIM1 and Pto_HIM1, respectively) and P. syringae pv. *actinidiae* biovar 3 grown in the presence of kiwifruit (Psa3_HIMK1) or tomato (Psa3_HIMT1) extracts. Clusters were generated using MeV with normalized fluorescence values, and blue triangles indicate the different clusters.

To determine whether the genes induced by kiwifruit extract after 1 h are specifically associated with the presence of plant signals, we compared these genes to those induced after 4 h in the apoplast-like medium with (HIMK4) or without (HIM4) host plant extract. The resulting Venn diagram shows that 68% (49/72) of the genes induced by kiwifruit extract after 1 h were still induced after 4 h (HIMK4) compared to 1 h in minimal medium (HIM1) (Fig. 2A), and 43 of them were also induced after 4 h in the absence of kiwifruit extract (HIM4 versus HIM1; Data Set S4). Moreover, compared to 1 h in the apoplast-like medium (HIM1), 121 genes were upregulated in the presence and absence of kiwifruit extract, representing ~70% (HIMK4 versus HIM1) and ~92% (HIM4 versus HIM1) of the induced genes, respectively. Hierarchical clustering of all DEGs identified under these conditions revealed three major clusters with different profiles (Fig. 2B). In cluster I, transcript levels were modulated specifically in the presence of kiwifruit extract after 1 h. In cluster II, transcript levels increased after 4 h regardless of the presence or absence of kiwifruit extract. Finally, the larger cluster, cluster III, contained genes that were induced in the presence of kiwifruit extract after 1 h, followed by a further increase in expression after 4 h regardless of the presence or absence of the kiwifruit extract. Interestingly, although gene ontology enrichment revealed no significant categories in clusters I or II, the genes in cluster III were significantly enriched for protein transport/secretion functions involving the T3SS (Fig. 2B, right), suggesting that kiwifruit signals do not induce T3SS (and related effectors) but rather anticipate the upregulation that would normally occur after 4 h in minimal medium. In line with this hypothesis, no DEGs were identified in P. syringae pv. *actinidiae* biovar 3 following incubation for 4 h in the presence or absence of kiwifruit extract, confirming that the genes were still induced under apoplast-like conditions but only after a delay.

Further comparison of T3SS-related gene expression in P. syringae pv. *actinidiae* biovar 3 with that in the model bacterium P. syringae pv. *tomato* DC3000 revealed that almost all *hrp*/*hrc* genes in the former (24/27) were induced by incubation in minimal medium for 1 to 4 h, whereas none were induced in P. syringae pv. *tomato* DC3000 over the same period (Data Set S4). Moreover, after 1 h in HIM, several *hrp*/*hrc* genes in P. syringae pv. *actinidiae* biovar 3 appeared to be downregulated compared to P. syringae pv. *tomato* DC3000 (Fig. 2C). This indicated that transcription levels were already elevated in P. syringae pv. *tomato* DC3000 after incubation for 1 h in HIM, whereas the same levels in P. syringae pv. *actinidiae* biovar 3 were only reached several hours later. This suggests the presence of a slower T3SS induction mechanism in P. syringae pv. *actinidiae* than in the model strain P. syringae pv. *tomato* DC3000, which was supported by the hierarchical clustering of *hrp*/*hrc* genes showing that the transcript level of most genes (cluster I) was higher overall in P. syringae pv. *tomato* DC3000 (Pto_HIM1) than in P. syringae pv. *actinidiae* biovar 3 (Psa3_HIM1), with only a few genes in cluster II showing different behavior (Fig. 2D). The faster response of P. syringae pv. *tomato* DC3000 to minimal medium was also supported by the very small number of genes modulated in this strain after incubation for 1 h in the presence of kiwifruit or tomato extracts, or incubation for 1 to 4 h in the apoplast-like medium (Data Sets S5 to S7).

Interestingly, the expression level of *hrp*/*hrc* genes in P. syringae pv. *actinidiae* biovar 3 was similar to or higher than that in P. syringae pv. *tomato* DC3000 when grown in the presence of the kiwifruit extract, with 20/27 genes showing a significantly higher expression level in P. syringae pv. *actinidiae* biovar 3. This supports the presence of kiwifruit-derived signals that trigger P. syringae pv. *actinidiae* biovar 3 to induce the rapid expression of T3SS-related genes, reaching or exceeding the transcript levels observed in P. syringae pv. *tomato* DC3000 but at an earlier stage (Fig. 2C). A similar trend was observed in the presence of tomato extract, although the genes were modulated to a lesser extent than in the presence of the kiwifruit extract (Fig. 2D). Overall, these data show that T3SS induction is faster in P. syringae pv. *tomato* DC3000 than in P. syringae pv. *actinidiae* biovar 3 under apoplast-like conditions but that plant signals can anticipate T3SS activation in P. syringae pv. *actinidiae* biovar 3.

### Protein secretion by P. syringae pv. *actinidiae* biovar 3 is enhanced by plant extracts *in vitro*.

**FIG 3:**
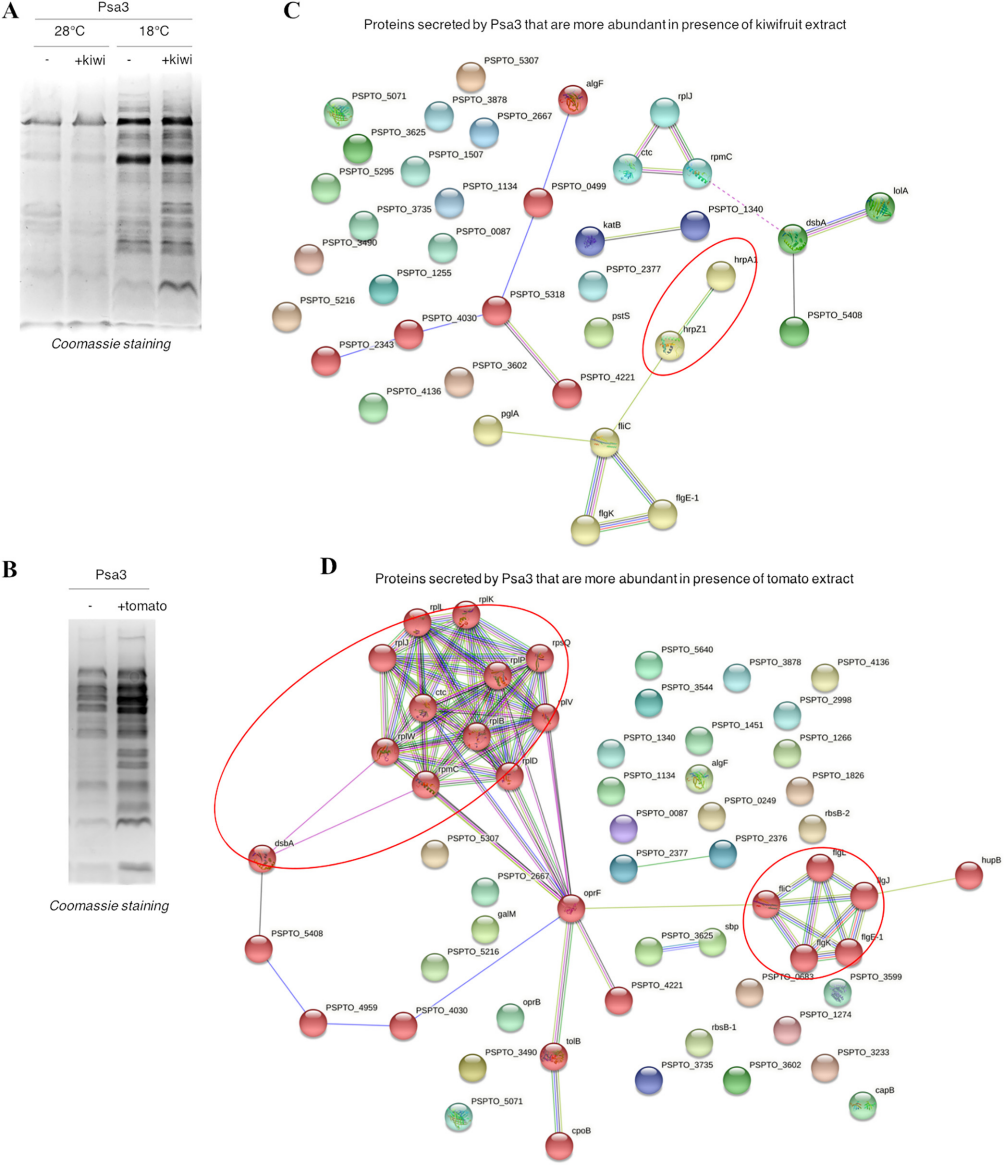
Kiwifruit and tomato extracts increase P. syringae pv. *actinidiae* biovar 3 (Psa3) protein secretion *in vitro* in a temperature-dependent manner. (A) Comparison of the *in vitro* secretomes of P. syringae pv. *actinidiae* biovar 3 grown in minimal medium (HIM) with or without kiwifruit extract at 28 or 18°C. (B) Comparison of the *in vitro* secretomes of P. syringae pv. *actinidiae* biovar 3 grown in minimal medium (HIM) with or without tomato extract at 18°C. (C and D) Functional relationships among proteins secreted by P. syringae pv. *actinidiae* biovar 3 in the presence of kiwifruit (C) or tomato (D) extracts, analyzed using STRING.

To determine whether the fast response of P. syringae pv. *actinidiae* biovar 3 to plant signals correlates with T3SS functionality, we analyzed the profile of the proteins secreted *in vitro*. Initially, we tested the bacteria at 28°C, the optimal growth temperature used for transcriptomic analysis in this study and previous reports (4). Surprisingly, although many genes related to protein secretion were induced in minimal medium at this temperature, very few proteins overall were detected in the extracellular medium, regardless of the presence or absence of kiwifruit extract (Fig. 3A). Interestingly, a greater number of secreted proteins were detected at 18°C, close to the optimal infection temperature for P. syringae pv. *actinidiae*. Furthermore, in line with the upregulation of T3SS-related genes described above, the number of secreted proteins and the overall signal intensity increased in the presence of kiwifruit extract. Similarly, the kiwifruit extract significantly increased the number of proteins secreted by P. syringae pv. *actinidiae* biovar 1 at 18°C (Fig. S2, left), in line with the greater *hrpA1* promoter activity reported in response to kiwifruit extract in this biovar (4). In contrast, the protein profile secreted by biovar 2 featured a larger number of secreted proteins at 28°C and showed no response to the kiwifruit extract (Fig. S2, right), in accordance with the different behavior of biovar 2, reflecting its inability to induce the T3SS (4). Given that *hrpA1* promoter activity was not influenced by temperature (Fig. S3), the observed temperature-dependent protein secretion may be related to secretion system assembly or functionality. Likewise, the addition of tomato extract significantly increased the total number of proteins secreted by P. syringae pv. *actinidiae* biovar 3 compared with that in cells incubated in minimal medium at 18°C (Fig. 3B). Additional analysis of P. syringae pv. *actinidiae* biovar 3 growth under different conditions revealed that temperature did not influence the growth rate (comparing the growth in HIM at 18°C and 28°C) (Fig. S4). Similarly, the addition of kiwifruit extract did not significantly change the growth, thus supporting the significance of protein abundance increase under such conditions. Conversely, the presence of tomato extract slightly increased the cell density but only when the incubation was performed at 28°C. These results thus confirm that the differences observed at the protein level are attributable to the secretion efficiency depending on culture conditions rather than a higher growth rate.

We identified 195 secreted proteins that differed in abundance based on secretome analysis by mass spectrometry (Data Sets S8 and S9). Semiquantitative proteomic analysis showed that the abundance of 81 proteins was significantly modulated in the presence of kiwifruit extract (33 up and 48 down), and 114 proteins were similarly affected in the presence of tomato extract (56 up and 58 down) in each case compared to in HIM, with 67 proteins affected by both extracts (Fig. S5). Potential functional interactions among these proteins were investigated using STRING (Fig. 3C and D). Kiwifruit extract significantly increased the secretion of HrpA1 (fold change [FC], +6.47) and HrpZ1 (FC, +2.29), two of the three most abundant harpins secreted by the T3SS involved in effector transport (14). These proteins were included in a cluster with proteins involved in the flagellum-related T3SS and the flagellin protein FliC (Fig. 3C, red cluster). Interestingly, the latter were also secreted more abundantly in the presence of tomato extract (Fig. 3D, green cluster). Moreover, in line with the transcriptome profile, the tomato extract induced the secretion of proteins related to translation (yellow cluster) and proteins involved in the Pal-Tol system, which plays a role in the T3SS and flagellum-mediated virulence in enterohemorrhagic Escherichia coli (15). Notably, both extracts also induced the secretion of the disulfide bond-forming protein DsbA, which is required for the T3SS to function in P. syringae pv. *tomato* DC3000 (16).

The kiwifruit extract did not modify the secreted protein profile in P. syringae pv. *tomato* or P. syringae pv. *actinidiae* biovar 2, while the abundance of proteins in both supernatants was increased in the presence of the tomato extract (Fig. S6A and B). Since this extract provided a signal when used alone as an input, this increase could be due in part to tomato proteins/peptides (Fig. S6C). Conversely, no signal was observed with the kiwifruit extract, confirming that protein increase was only due to bacterial secretion.

Regarding the detection of ribosomal proteins in the extracellular medium, a major cell lysis, although not ruled out, appears very unlikely, since it should occur randomly among the different samples and would not lead to a statistically significant increase in ribosomal protein abundance only in the presence of plant extracts. As an alternative, the presence of such proteins, enriched only in the secretomes of bacteria treated with plant extracts, may reflect another process related to protein secretion. Indeed, several examples of cytoplasmic proteins actively exported across the cytoplasmic membrane, including ribosomal proteins themselves, have been reported previously in different bacterial species, including Helicobacter pylori, Staphylococcus aureus, and Listeria monocytogenes (17). Thus, a similar mechanism, also proposed in P. syringae pv. *tomato* (18), could thus be activated in the presence of plant extracts in P. syringae pv. *actinidiae* biovar 3 and may deserve further investigation.

### Earlier T3SS induction by plant signals influences the triggering of hypersensitive cell death by P. syringae pv. *actinidiae* biovar 3 in *A. thaliana*.

**FIG 4:**
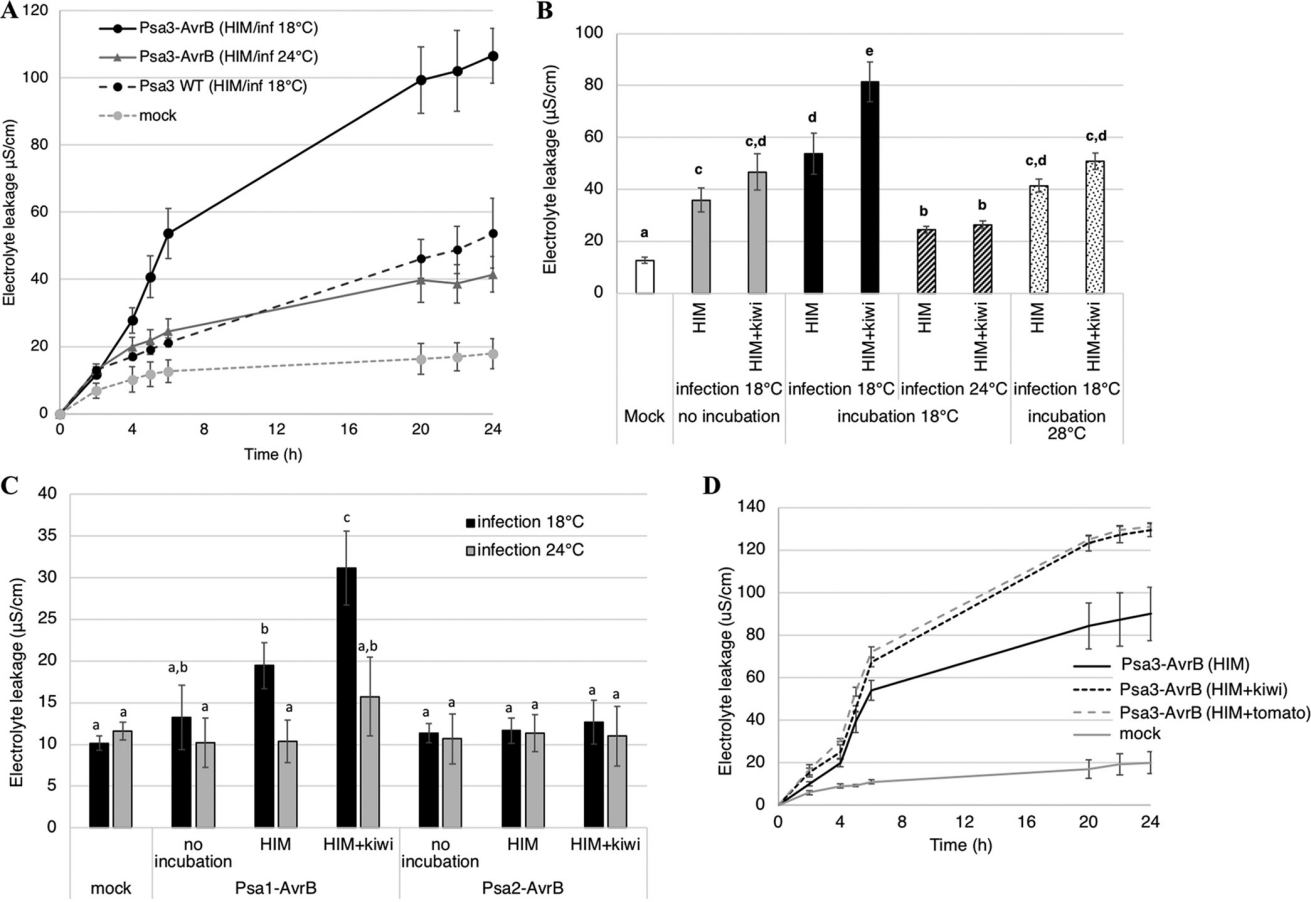
P. syringae pv. *actinidiae* biovar 3 preincubated under apoplast-like conditions with kiwifruit extract induces temperature-dependent hypersensitive cell death in the nonhost plant Arabidopsis thaliana Col-0. (A) Time course of electrolyte leakage in *A. thaliana* Col-0 leaf disks infiltrated with P. syringae pv. *actinidiae* biovar 3 (Psa3) wild type (WT) or carrying the *avrB* gene (Psa3-AvrB) and preincubated in minimal medium (HIM) prior to infiltration. The infected leaf disks were incubated at 18 or 24°C. Mock-infiltrated leaf disks were used as negative controls. Values correspond to the mean of three biological replicates, each including three technical replicates, plus or minus standard error. (B and C) Electrolyte leakage 6 h postinfiltration in *A. thaliana* Col-0 leaf disks inoculated with (B) P. syringae pv. *actinidiae* biovar 3 or (C) biovars 1 or 2, carrying the *avrB* gene preincubated (or not) for 1 h in HIM or in the presence of kiwifruit extract (HIM+kiwi). The bacteria were preincubated at (B) 18 or 28°C or (C) 18°C, and the infected leaf disks were incubated at 18 or 24°C, as indicated. Values correspond to the mean of three biological replicates, each including three technical replicates, plus or minus the standard error. Lowercase letters indicate statistically significant differences according to analysis of variance (ANOVA; *P* < 0.05). (D) Time course of electrolyte leakage in *A. thaliana* Col-0 leaf disks infiltrated with P. syringae pv. *actinidiae* biovar 3 carrying the *avrB* gene and preincubated for 1 h in HIM or HIM supplemented with kiwifruit (HIM+kiwi) or tomato (HIM+tomato) extracts prior to infiltration. The bacteria were preincubated at 18°C, and leaf disks were incubated at the same temperature. Mock-infiltrated leaf disks were used as negative controls. Values correspond to the mean of three biological replicates, each including three technical replicates, plus or minus the standard error.

Since kiwifruit is its native host plant, P. syringae pv. *actinidiae* biovar 3 is already able to inject type III effectors, which are responsible for either its virulence, as demonstrated using the Δ*hrcC* mutant, which lost its virulence on the highly susceptible *Actinidia chinensis* var. *chinensis* Hort16A, or hypersensitive response (HR) induction in some resistant kiwifruit cultivars (19). Conversely, P. syringae pv. *actinidiae* biovar 3 is unable to induce a hypersensitive response in *A. thaliana* ecotype Col-0 (16) (Fig. S7A), even though it produces the AvrRpm1 effector recognized by the host receptor RPM1. However, this does not reflect the inability of AvrRpm1_Psa_ to trigger an RPM1-dependent response (20), because a transformed P. syringae pv. *actinidiae* biovar 3 strain carrying the well-known avirulence factor AvrB (Psa3-AvrB) still failed to induce hypersensitive cell death at 24°C, the typical temperature used for ion leakage experiments, or at 18°C, which is required for efficient protein secretion by P. syringae pv. *actinidiae* biovar 3 (Fig. S7B). Thus, *A. thaliana* Col-0 represented an interesting model to investigate the effect of T3SS induction anticipation by plant signals. Interestingly, the preincubation of Psa3-AvrB, and to a lesser extent of P. syringae pv. *actinidiae* biovar 3 wild type, in HIM for 1 h before infiltration in *A. thaliana* Col-0 led to the induction of a significant hypersensitive response, which was enhanced if kiwifruit extract was present in the preincubation medium (Fig. 4A and B). This demonstrates that induction of the T3SS under apoplast-like conditions and its enhancement by kiwifruit extract, at the mRNA and protein levels, leads to a functional T3SS that can efficiently translocate effectors into *A. thaliana* Col-0. Like protein secretion *in vitro*, the hypersensitive response induced by Psa3-AvrB is strictly temperature dependent, occurring only when infected leaf disks are incubated at 18°C. In contrast, the preincubation temperature does not prevent Psa3-AvrB from triggering the hypersensitive response (Fig. 4B). Similarly, P. syringae pv. *actinidiae* biovar 1 carrying the avirulence factor AvrB (Psa1-AvrB), preincubated for 1 h in HIM, induced a hypersensitive response in *A. thaliana* Col-0, enhanced in the presence of kiwifruit extract and in a temperature-dependent manner, like P. syringae pv. *actinidiae* biovar 3 (Fig. 4C). Remarkably, however, preincubation did not allow Psa2-AvrB to trigger a hypersensitive response, regardless of the temperature of infection, showing that the hypersensitive cell death induced by P. syringae pv. *actinidiae* biovar 3 (and biovar 1) is linked to the activation or enhancement of the T3SS, which does not occur in biovar 2. Tomato extract also enhanced the hypersensitive response induced by biovar 3 (Fig. 4D), agreeing with the induction of the T3SS at the mRNA level. However, although treatment with tomato extract also increased the number of proteins in P. syringae pv. *tomato* DC3000 and P. syringae pv. *actinidiae* biovar 2, it did not enhance the hypersensitive response induced by the same strains carrying AvrB (Fig. S6 and S8). This confirms that the enhanced hypersensitive response triggered by P. syringae pv. *actinidiae* biovar 3 is dependent on the ability of plant extracts to stimulate the T3SS.

## DISCUSSION

The role of host plant signals in the induction of P. syringae pv. *actinidiae* virulence is not completely understood, and the early phase of the bacterial response has not been studied in detail. We therefore profiled the transcriptome of P. syringae pv. *actinidiae* biovar 3 incubated in minimal medium with and without kiwifruit or tomato leaf extracts for 1 h. The presence of the kiwifruit extract led to the specific and strong induction of genes related to the T3SS, particularly the syringe structure (helper and secretion proteins), after only 1 h, suggesting that the host interaction anticipates T3SS stimulation. The tomato extract also induced *hrp* gene expression, but the response was less intense compared to that for the kiwifruit extract. Our results highlight the capacity of host plants to trigger the early activation of the T3SS. In line with the fact that the bacteria were already incubated in apoplast-like conditions, which induce virulence gene expression (4), only a few genes were found further differentially regulated by plant extracts. This reinforces the specificity of the effect of induction anticipation by plant signals, since the small changes observed were very relevant, demonstrating that plant signal(s) do not lead to complete transcriptome reprogramming but rather contribute to better induction of a small subset of genes required for bacteria to efficiently infect plants. This indicates that the molecular analysis of T3SS induction may provide important information about the capacity of a bacterial strain to infect plants, even if a natural infection has yet to be reported.

Despite the presence of at least 30 effectors per strain and many pathovars, P. syringae pv. *actinidiae* still appears to have a limited host range. For example, the disease caused by P. syringae pv. *actinidiae* develops slowly in *A. thaliana*, if at all (21). Moreover, effector-triggered immunity (ETI) in *A. thaliana* is pervasive following inoculation with P. syringae pv. *actinidiae*, because 97% of these strains carry one or more immunity-eliciting effectors, and 95% are recognized by the host due to two prominent R proteins (ZAR1 and CAR1). This demonstrates why the presence/absence of effectors and/or R proteins is necessary but not sufficient to predict the outcome of interactions between plants and bacteria. Indeed, P. syringae pv. *actinidiae* possesses the effector HrpZ5, which induces a hypersensitive response in *A. thaliana* Col-0 cells when introduced by P. syringae pv. *tomato* DC3000 but not by P. syringae pv. *actinidiae*, as shown here and in previous studies (16). Moreover, the genetic transformation of P. syringae pv. *actinidiae* with the well-characterized avirulence gene *avrB* does not significantly improve the induction of a hypersensitive response. Therefore, it appears that P. syringae pv. *actinidiae* is likely unable to transfer its effectors efficiently, at least in *A. thaliana* Col-0. Interestingly, the 1-h preincubation of P. syringae pv. *actinidiae* cells in minimal medium allowed the induction of a hypersensitive response by the modified Psa3-AvrB strain, which was enhanced by adding kiwifruit extract to the medium. According to transcriptomic data, the only event occurring in this very short time frame was the upregulation of the T3SS in the presence of plant extracts. Thus, these small differences are responsible for the observed phenotype, i.e., the capacity of P. syringae pv. *actinidiae* biovar 3 to exploit its T3SS in an otherwise nonhost plant, demonstrating that the enhancement of T3SS induction prior to the infiltration of *A. thaliana* Col-0 allows the assembly of a functional T3SS that can transfer effectors recognized by the corresponding R proteins and thus induce an efficient hypersensitive response.

As in a real dialogue, the interaction between pathogens and plants involves the mutual sensing of signals followed by appropriate responses. According to the current zig-zag model, plants first develop a general response based on pathogen-associated molecular patterns (PAMPs), known as PAMP-triggered immunity (PTI), to inhibit microbial colonization of the apoplast. Successful bacterial pathogens counter this response by using the T3SS to introduce PTI-suppressing type III effectors (22–24), which were acquired long before the advent of agriculture and even before the speciation of some of their host plants (25). However, PTI can directly or indirectly inhibit the introduction of type III effectors (26), and when induced by flagellin in nonhost plants, PTI can suppress the hypersensitive response elicited by P. syringae pv. *averrhoi* (27). Virulent bacteria must rapidly overcome T3SS restriction by PTI, allowing them to transfer their effectors and initiate effector-triggered susceptibility (ETS). In compatible interactions (which lead to the expression of disease symptoms), the initially low quantity of effectors transferred to the plant may suppress PTI enough to allow the transfer of further effectors (26). However, this would mean that the failure of avirulent bacteria would rely on the absence of effectors that can block PTI, which is unrealistic considering the large effector repertoire of phytopathogenic bacteria. It is therefore reasonable to assume that the timing of bacterial virulence and plant defense responses may be another fundamental parameter governing the outcome of such interactions. In agreement with this hypothesis, three pathovars of Pseudomonas savastanoi (namely, P. savastanoi pv. *fraxinii*, P. savastanoi pv. *savastanoi*, and P. savastanoi pv. *nerii*) showed different expression profiles for the four main T3SS-related genes when incubated in minimal medium, possibly accounting for the differences in their virulence (28). This may play a role in the ability of pathogenic strains to overcome PTI-mediated T3SS inhibition and inject virulence effectors that lead to the development of disease symptoms, as shown for the P. syringae pv. *tomato* DC3000/*A. thaliana* and P. syringae pv. *tabaci*/tobacco interactions. Accordingly, we found that P. syringae pv. *tomato* DC3000 was able to fully activate its T3SS within 1 h. Indeed, analysis conducted as early as 1 h showed that the P. syringae pv. *tomato* DC3000 cells induced T3SS much more quickly than did the P. syringae pv. *actinidiae* biovar 3 cells, thus highlighting differences in T3SS activation among different Pseudomonas syringae strains, as already observed between different P. syringae pv. *actinidiae* biovars (4). It is tempting to speculate that such differences in T3SS induction timing may, at least in part, contribute to the diverse range of hosts that P. syringae strains may infect (29).

As well as inducing T3SS-related gene expression, the plant extracts also appear to act at the protein level by stabilizing some T3SS components, such as HrpZ1. Indeed, the kiwifruit and tomato extracts both increased the abundance of DsbA without affecting *dsbA* mRNA levels. A similar correlation was observed in *A. thaliana* mutants with a modified salicylic acid (SA) pathway during interactions with P. syringae pv. *tomato* DC3000, resulting in higher levels of HrpZ1 and DsbA after 24 and 48 h but no change in *dsbA* gene expression (12). Interestingly, a P. syringae *dsbA* mutant was less virulent and showed partial impairment of the T3SS (30). Protein secretion through the T3SS still occurs in this background, but the efficiency is lower, as also observed in a *dsbA* mutant strain of Yersinia pestis (31). The accumulation of DsbA in response to plant extracts may therefore contribute to the higher efficiency of protein secretion through the T3SS under the same conditions that induce T3SS-related gene expression.

We found evidence that temperature is a key factor governing the balance between virulence and plant defense. Two recent studies revealed transcriptomic and proteomic differences between virulent and avirulent forms of P. syringae pv. *tomato* DC3000 during interactions with *A. thaliana* (12). However, the experimental design did not consider the role of temperature in the outcome, regardless of the presence/absence of avirulence factors. Nevertheless, the role of temperature has been described, and it clearly regulates the efficiency of defense responses in plants (32, 33). Based on our hypothesis, in which temperature is a key regulator of the T3SS, we used the Psa3-AvrB strain to monitor the induction of a hypersensitive response in *A. thaliana* Col-0 and found that the temperature of infection was more important than the temperature of preincubation for T3SS induction and thus T3SS functionality. In this pathosystem, programmed cell death is triggered at 18°C, suggesting that the P. syringae pv. *actinidiae* T3SS is more efficient than the PTI response (32), and our analysis of *in vitro* protein secretion provided supportive evidence. Interestingly, the hypersensitive response triggered by P. syringae pv. *tomato* DC3000 is not temperature dependent. However, high temperatures promote P. syringae pv. *tomato* DC3000 effector translocation, probably by inhibiting the SA-mediated pathway (34), in line with the greater abundance of HrpZ1, HrpK1, and HrpW in *A. thaliana sid2*/*pad4* mutants lacking this pathway (12). However, the more efficient translocation of type III effectors, which could contribute to a stronger hypersensitive response, would be balanced by the lower capacity of the plant to induce a hypersensitive response in the absence of SA-mediated signals (35). In the same manner, the production of the phytotoxin coronatine by P. syringae pv. *tomato* DC3000 is not temperature dependent, but in P. syringae pv. *glycinea*, it is induced only at low temperatures (36). Overall, this highlights the peculiar behavior of P. syringae pv. *tomato* DC3000, which differs at least from P. syringae pv. *actinidiae* biovar 3 and P. syringae pv. *glycinea* by inducing virulence regardless of the temperature. In this context, it would be interesting to evaluate the dynamic T3SS activation process in different strains of the Pseudomonas complex to determine whether this may affect the outcome of the interactions with different plant species, depending on the capacity of plants to induce rapid defense responses that restrict the T3SS before its induction.

Our work demonstrates the need to consider not only the presence/absence of a bacterial effector repertoire, combined with the presence/absence of the corresponding targets/*R* genes in the host plant (leading to susceptibility and disease, respectively), but also the capacity (or opportunity) for plants to activate their basal defense mechanisms or for bacteria to express and assemble an efficient T3SS for the injection of virulence effectors. In this scenario, the aim of the arms race between plants and bacteria is not only to evolve new weapons (i.e., the acquisition of new genomic features) but also to gain the capacity to mobilize the existing ones more rapidly, thus mounting an effective defense (plants) or enhancing virulence (bacteria). The temperature would play a key role in this competition, suggesting that the outcome of the molecular interaction with the plant is highly contingent on the biotic and abiotic context. Overall, our findings may explain, at least in part, the overlapping host range continuum for strains of the P. syringae pv. *actinidiae* complex (29).

## MATERIALS AND METHODS

### Bacterial strains and media.

Single colonies of Pseudomonas syringae pv. *actinidiae* J35 (biovar 1), KN.2 (biovar 2), and CRA-FRU 8.43 (biovar 3) and Pseudomonas syringae pv. *tomato* DC3000 grown on a rich solid medium (King’s B [KB] agar) were inoculated into KB medium and incubated overnight at 28°C, with shaking at 200 rpm. When cells reached the late log phase, they were collected by centrifugation (5,000× *g*, 10 min, room temperature), washed three times in liquid *hrp*-inducing medium (HIM) (8), and resuspended at a final optical density at 600 nm (OD_600_) of 0.1 in HIM with or without kiwifruit or tomato leaf extract at a final concentration of 1%. After incubation for different times at 18°C or 28°C (as indicated in the manuscript), with shaking at 200 rpm, the cells were harvested by centrifugation as above, and the pellets (~2.4 × 10^9^ cells; for transcriptomic analyses) or the supernatants (for proteomic analyses) were stored at −20°C or −80°C, respectively.

### Preparation of leaf extracts.

Micropropagated plantlets of *A. chinensis* var. *deliciosa* ‘Hayward’ and Solanum lycopersicum were transferred to small soil-containing pots grown in a climate-controlled room at 24°C with 60% relative humidity and a 16-h/8-h light/dark cycle. Leaves and petioles were removed from 2- to 4-month-old kiwifruit or tomato plants (*n* = 20), weighed, ground in a kitchen juice extractor, and squeezed onto ice (using a ratio of 1/5 [wt/vol]). The collected raw extracts were centrifuged repeatedly (5,000 rpm, 10 min, 4°C) until clarified, and the resulting supernatants were passed through a 0.2-μm sterilization filter before aliquoting and storing at −20°C until use.

### RNA extraction and microarray chip hybridization.

RNA was extracted from each sample using the Spectrum plant total RNA kit (Sigma-Aldrich). Residual DNA was removed using the TURBO DNA-free kit (Thermo Fisher Scientific, Waltham, MA, USA). RNA concentrations were determined using a NanoDrop 2000 spectrophotometer (Thermo Fisher Scientific). First-strand cDNA was synthesized from 1 μg total RNA using the SuperScript III reverse transcriptase enzyme kit (Invitrogen, Carlsbad, CA, USA) and random hexadeoxynucleotides (Promega, Madison, WI, USA). Samples were labeled for microarray analysis using the One-Color microarray-based gene expression analysis low-input Quick Amp whole-transcript (WT) labeling kit (Agilent Technologies, Santa Clara, CA, USA), according to the manufacturer’s instructions.

### Microarray and data analysis.

The bacterial transcriptome was interrogated using a custom SurePrint G3 GE 8 × 60K chip, designed in-house and produced by Agilent Technologies (4). The fluorescence intensity for each probe was measured using an Agilent G4900DA SureScan microarray scanner system with Agilent Scan Control software, and the data were extrapolated using Agilent Feature Extraction software. Fluorescence intensities were calculated by robust multiarray averaging, including adjustment for background intensity, log_2_ transformation, and quantile normalization. Normalized data were used to identify differentially expressed genes (DEGs) with threshold values of *P* < 0.05 and log_2_ FC values of >|1|. DEGs were compared across different strains and/or conditions using the online software Calculate and Draw Custom Venn Diagrams (http://bioinformatics.psb.ugent.be/webtools/Venn/).

### Measurement of hypersensitive cell death in *A. thaliana*.

Hypersensitive cell death was assessed by measuring electrolyte leakage from infected cells (37). Bacterial strains carrying the avirulence gene *avrB* were grown on KB agar and single colonies were used to inoculate 30 mL of KB liquid medium supplemented with 50 μg/mL rifampicin (Pto-AvrB only) and 50 μg/mL kanamycin (all strains). After overnight incubation at 28°C, with shaking at 200 rpm, the suspensions were centrifuged (5,000 rpm, 15 min, room temperature), and the pellets were washed three times with 10 mM MgCl_2_. The bacteria were resuspended in 10 mM MgCl_2_, HIM or HIM + 1% kiwi (final volume, 20 mL) at an OD_600_ value of 0.1. Three 8-mm leaf disks (representing three different leaves) each from six *A. thaliana* Columbia-0 (Col-0) plants were vacuum infiltrated with the bacterial suspensions or with 10 mM MgCl_2_, HIM, or HIM plus kiwi as the negative controls. All infiltrated leaf disks were washed in Milli-Q water for 30 min with gentle agitation (90 rpm) and distributed among three wells (six disks/well), each containing 2 mL of Milli-Q water. The three wells acted as three technical replicates for each condition. Conductivity (μS/cm) was measured after 0, 2, 4, 6, and 24 h. For each time point and each condition, conductivity was calculated as the average of the values of the three technical replicates, and the values at time zero were subtracted for normalization.

### Analysis of *hrpA1* promoter activity.

P. syringae pv. *actinidiae* strain CRAFRU8.43 carrying the p*hrpA1*::*gfp* construct (38) was grown on KB agar, and single colonies were used to inoculate 15 mL KB liquid medium supplemented with 40 μg/mL gentamicin, followed by overnight incubation at 28°C, with shaking at 200 rpm. The cells were recovered by centrifugation (5,000 rpm, 15 min, room temperature) and were resuspended in fresh HIM with or without kiwifruit or tomato leaf extract (1% final concentration) at an OD_600_ value of 0.1. We transferred 200 μL of bacterial cells to the wells of transparent 96-multiwell plates, and GFP fluorescence was measured every 15 min for 8 h using an Infinite 200 Pro fluorimeter (Tecan, Männedorf, Switzerland) at an excitation wavelength (λ_ex_) of 485 nm and an emission wavelength (λ_em_) of 535 nm. The fluorescence value at time zero was subtracted from the values at other time points for normalization.

### Hierarchical clustering.

Normalized fluorescence intensities from microarray experiments were imported as data matrices into MeV (39). The data were adjusted as median center genes/rows and clustered using the hierarchical clustering module. Gene and sample trees were clustered with optimized gene and sample leaf orders using Pearson’s correlation and average linkage clustering. The trees were subsequently cut into clusters using a distance threshold (0.5 to 1) empirically adjusted to highlight the most relevant features of the trees.

### Collection of secreted proteins and SDS-PAGE analysis.

The bacterial strains were grown on KB agar, and single colonies were used to inoculate 50 mL of KB liquid medium, followed by overnight incubation at 28°C, with shaking at 200 rpm. The cell suspensions were centrifuged (5,000 rpm, 10 min, room temperature), and the pellets were washed three times in HIM before resuspension in 50 mL HIM with or without kiwifruit or tomato extract (final concentration, 1%) at a final OD_600_ value of 0.8. The bacteria were incubated for 24 h at 18 or 28°C, with shaking at 200 rpm. Following another round of centrifugation as above, the supernatants were concentrated 50-fold using Amicon Ultra-4 centrifugal filters with a molecular weight cutoff of 3 kDa (Merck-Millipore, Burlington, MA, USA). Samples were mixed with 3× Laemmli loading buffer and heated for 3 min at 85°C before SDS-PAGE analysis. Polyacrylamide gels were stained with Coomassie brilliant blue R-250 for protein visualization.

### Analysis of secreted proteins by LC-MS/MS.

Supernatants obtained as described above were precipitated overnight at −20°C with four volumes of ice-cold acetone. After centrifugation (14,000 rpm, 10 min, 4°C), the pellets were air-dried for 10 min and resuspended in solubilization solution comprising 7 M urea, 2 M thiourea, 3% (wt/vol) CHAPS, 40 mM Tris, and Complete Mini protease inhibitor cocktail (Roche, Basel, Switzerland). Proteins were quantified using the Bradford method (40). Before liquid chromatography-tandem mass spectrometry (LC-MS/MS) analysis, secreted proteins were digested with trypsin (Applied Biosystems, Waltham, MA, USA), as previously described (41). Comparative proteomic analysis by LC-MS/MS was carried out using a micro-LC device (Eksigent Technologies, Dublin, OH, USA) coupled to a TripleTOF 5600+ mass spectrometer (AB Sciex, Concord, ON, Canada). After peptide separation on a Halo fused C_18_ column (AB Sciex) using a 30-min gradient of acetonitrile in 0.1% formic acid, MS and MS/MS spectra were collected as previously described (42). Briefly, data-dependent analysis (DDA) was combined with cyclic data-independent analysis (DIA) using a 25-Da window (36 sweeps in total) for identification and label-free quantification. Following two DDA and three DIA acquisitions using Analyst TF v1.7 (AB Sciex), the DDA files were searched using Protein Pilot v4.2 (AB Sciex) and Mascot v2.4 (Matrix Science, Boston, MA, USA). Mascot was set to allow two missed cleavages, the instrument was set to electrospray ionisation quadrupole time-of-flight (ESI-QUAD-TOF) mode, and carbamidomethyl cysteine was specified as a fixed modification and oxidized methionine as a variable modification. Triplicate Sequential Window Acquisition of all Theoretical Mass Spectra scans were carried out for each sample, and the data were imported into Skyline for label-free quantification and the identification of secreted proteins. The peptide mass tolerance was set to 0.08 Da and the MS/MS tolerance to 10 ppm. The search was set to a monoisotopic mass with charges of 2^+^, 3^+^, and 4^+^. We searched a UniProt and Swiss-Prot reviewed database containing P. syringae pv. *actinidiae* group proteins, as well as a target-decoy database, with the false discovery rate set to 1%.

### Protein-protein interaction analysis.

Protein-protein interactions were predicted using the STRING database (http://string-db.org), including text mining (43). The species under investigation was set to a medium confidence level (0.400), and we retrieved known and predicted interactions. Additional white nodes and network depth were kept to the minimum value of 1 to exclude as many false-positive interactions as possible. Clusters were retrieved using Markov clustering (MCL), with the inflation parameter set to 1.1.

### Data availability.

The gene expression data sets generated by microarray for this study have been deposited in the NCBI GEO repository under the accession no. GSE211939. The mass spectrometry proteomics data have been deposited at the ProteomeXchange Consortium via the PRIDE partner repository with the data set identifier PXD036021.
