# Supplementary material for: Plant Signals Anticipate the Induction of the Type III Secretion System in Pseudomonas syringae pv. actinidiae, Facilitating Efficient Temperature-Dependent Effector Translocation
Source: Microbiol Spectr. 2022 Oct 26;10(6):e02073-22. doi: 10.1128/spectrum.02073-22 (PMC9770001; doi:10.1128/spectrum.02073-22)
Supplement: Supplemental file 1 — Captions to Data Sets S1 to S9; Fig. S1 to S8. Download spectrum.02073-22-s0001.pdf, PDF file, 1.9 MB [file spectrum.02073-22-s0001.pdf]

List of Supplementary Datasets and captions

Supplementary Dataset S1. List of genes differentially expressed in Psa3 grown for 1h in minimal medium supplemented with 1% kiwifruit extract (HIMK1) compared with minimal medium alone (HIM1).

Supplementary Dataset S2. List of genes differentially expressed in Psa3 grown for 1h in minimal medium supplemented with 1% tomato extract (HIMT1) compared with minimal medium alone (HIM1).

Supplementary Dataset S3. Comparison of genes differentially expressed in Psa3 grown for 1h in presence of 1% kiwifruit extract (HIMK1) or 1% tomato extract (HIMT1) compared with only minimal medium (HIM1)

Supplementary Dataset S4. List of genes differentially expressed in Psa3 grown for 4h in minimal medium (HIM4) compared with 1h in minimal medium (HIM1).

Supplementary Dataset S5. List of genes differentially expressed in Pto DC3000 grown for 1h in minimal medium supplemented with 1% kiwifruit extract (HIMK1) compared with minimal medium alone (HIM1).

Supplementary Dataset S6. List of genes differentially expressed in Pto DC3000 grown for 1h in minimal medium supplemented with 1% tomato extract (HIMT1) compared with minimal medium alone (HIM1).

Supplementary Dataset S7. List of genes differentially expressed in Pto DC3000 grown for 4h in minimal medium (HIM4) compared with 1h in minimal medium (HIM1).

Supplementary Dataset S8. List of proteins secreted by Psa3 that show a different abundance in presence of 1% kiwifruit extract compared with minimal medium alone.

Supplementary Dataset S9. List of proteins secreted by Psa3 that show a different abundance in presence of 1% tomato extract compared with minimal medium alone.

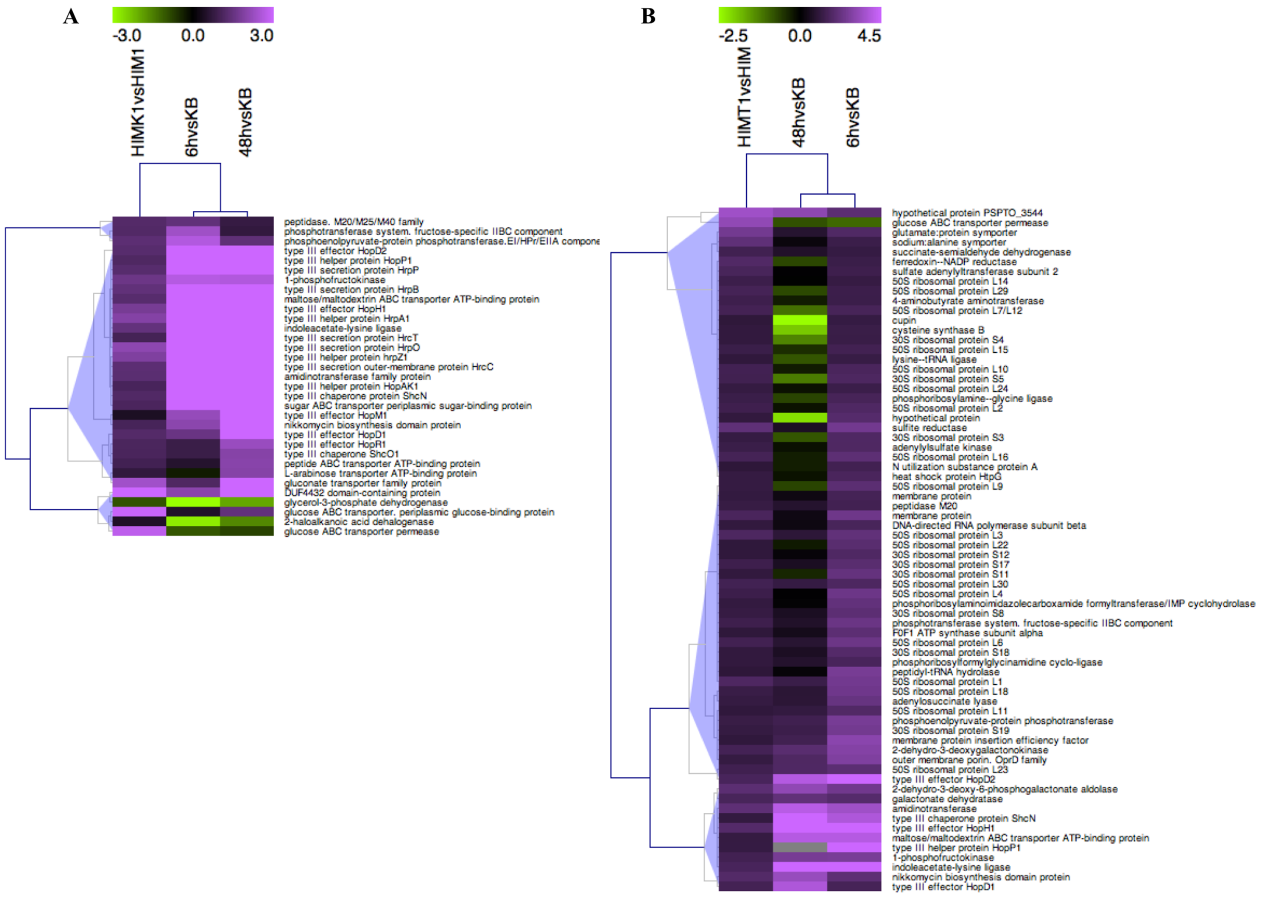

Supplementary Fig. S1. Hierarchical clustering of genes upregulated in *P. syringae* pv. *tomato* (Pto) DC3000 grown *in planta* for 6 or 48 h (Nobori et al., 2020) and *P. syringae* pv. *actinidiae* biovar 3 (Psa3) grown under apoplast-like conditions supplemented with (A) kiwifruit extract for 1 h (this work) or (B) tomato extract for 1 h (this work). Clusters were generated using MeV with normalized fluorescence values, and blue triangles indicate different gene clusters based on their expression profile.

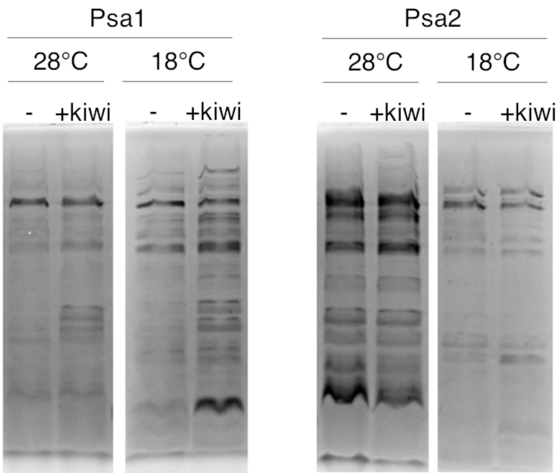

Supplementary Fig. S2. Profiles of proteins secreted *in vitro* by *P. syringae* pv. *actinidiae* biovar 1 (Psa1, left panel) or *P. syringae* pv. *actinidiae* biovar 2 (Psa2, right panel).

Bacterial cells were grown for 24 h at 18 or 28 °C in minimal (*hrp*-inducing) medium with or without kiwifruit extract. Polyacrylamide gels were stained with Coomassie Brilliant Blue.

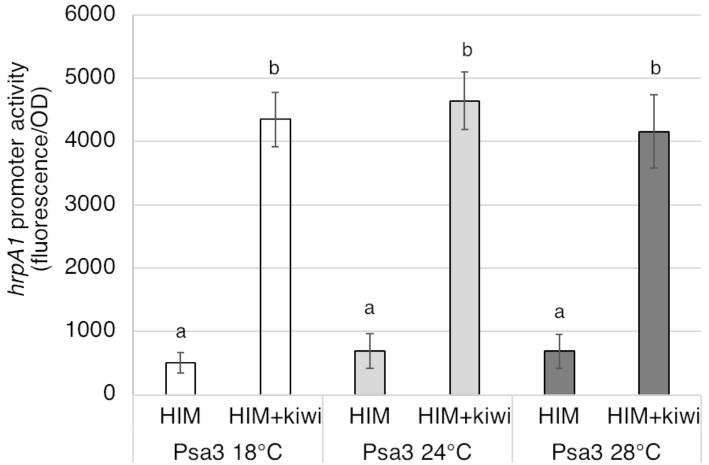

Supplementary Fig. S3. Activity of the *hrpA1* promoter in *P. syringae* pv. *actinidiae* biovar 3 (Psa3) grown at different temperatures in minimal medium with or without kiwifruit extract. Fluorescence values correspond to the mean of three biological replicates, each including three technical replicates, ±standard error. Different letters indicate statistically significant differences according to an analysis of variance (ANOVA) test ($p < 0.05$).

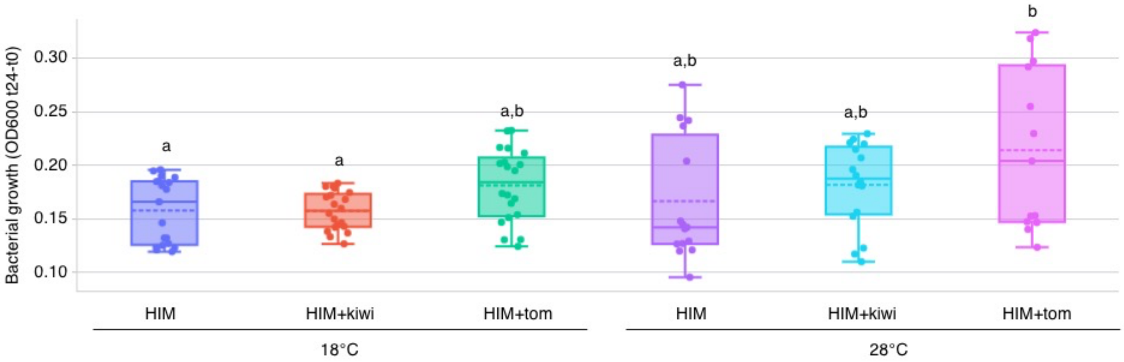

Supplementary Fig. S4. Effect of temperature and plant extract on *in-vitro* Psa3 growth in apoplast-like conditions.

Psa3 cells were grown for 24 h in minimal medium (*hrp*-inducing medium) at 18°C or 28°C, alone (HIM) or supplemented with kiwifruit (HIM+kiwi) or tomato (HIM+tom) extract at a final concentration of 1%. Bacterial density was evaluated by measuring the absorbance at 600nm using a a spectrophometer and the values in the boxplots represent the delta of absorbance between 0 and 24h. The values shown on the graphs have been obtained in four independent biological replicates. The different letters indicate a statistically significant difference among samples according to one-way ANOVA and post-hoc Tukey HSD test. Boxplots were performed using Chart Studio (https://chart-studio.plotly.com).

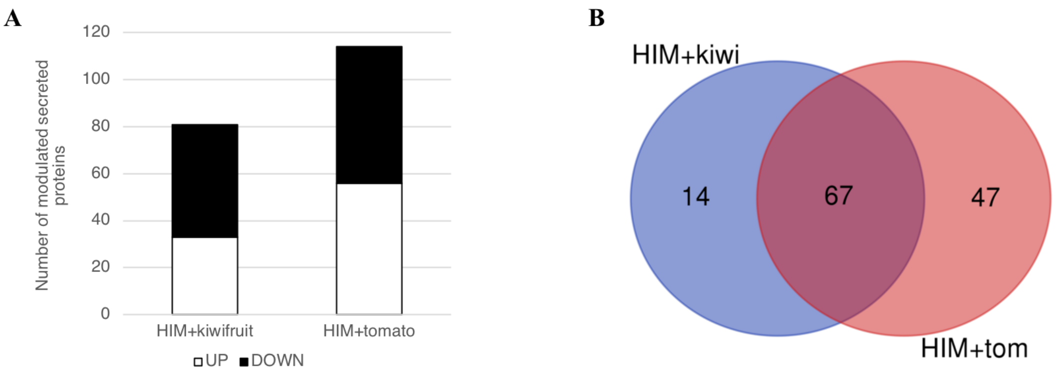

Supplementary Fig. S5. Analysis of *P. syringae* pv. *actinidiae* biovar 3 (Psa3) secreted proteins that differ in abundance following exposure to kiwifruit or tomato extracts.

(A) Number of secreted proteins that are more (UP, white bar) or less (DOWN, black bar) abundant in the Psa3 secretome following the incubation of cells in apoplast-like medium (HIM) containing kiwifruit (HIM+kiwifruit) or tomato (HIM+ tomato) extracts, relative to HIM alone. (B) Venn diagram showing the number of proteins that are more or less abundant in the Psa3 secretome specifically following exposure to kiwifruit or tomato extracts or common to both. The Venn diagram was generated using Draw Venn Diagram.

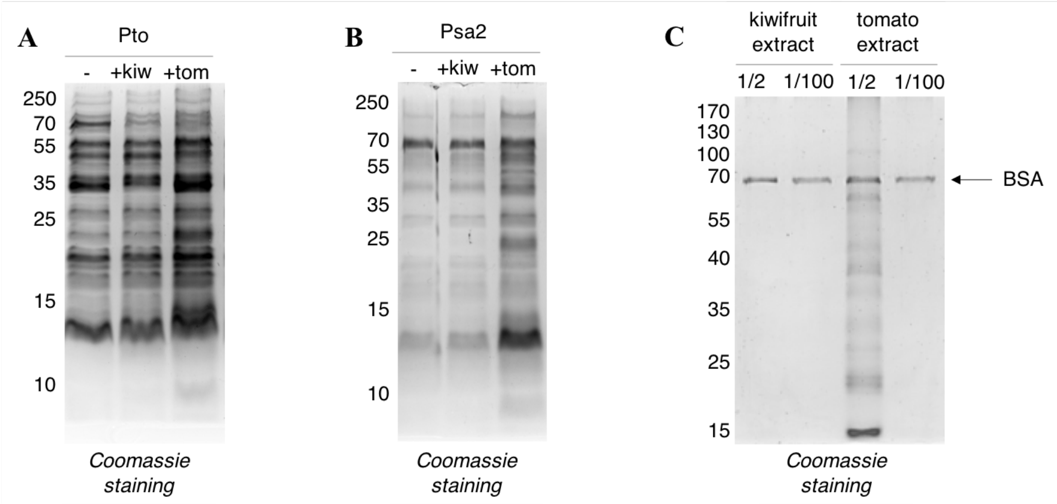

Supplementary Fig. S6. Comparison of the *in vitro* secretomes of *P. syringae* pv. *tomato* DC3000 or *P. syringae* pv. *actinidiae* biovar 2 in the presence of kiwifruit or tomato extracts.

(**A,B**) Bacterial cells of (A) Pto or (B) Psa2 were grown for 24 h at 18 °C in minimal (*hrp*-inducing) medium with and without kiwifruit (+kiwi) or tomato (+tom) extract. Supernatants were concentrated before analysis. (C) Pure kiwifruit and tomato extracts were loaded as inputs at two different concentrations. Bovine serum albumin (BSA) was added to the extracts as internal controls of the run. Polyacrylamide gels were stained with Coomassie Brilliant Blue.

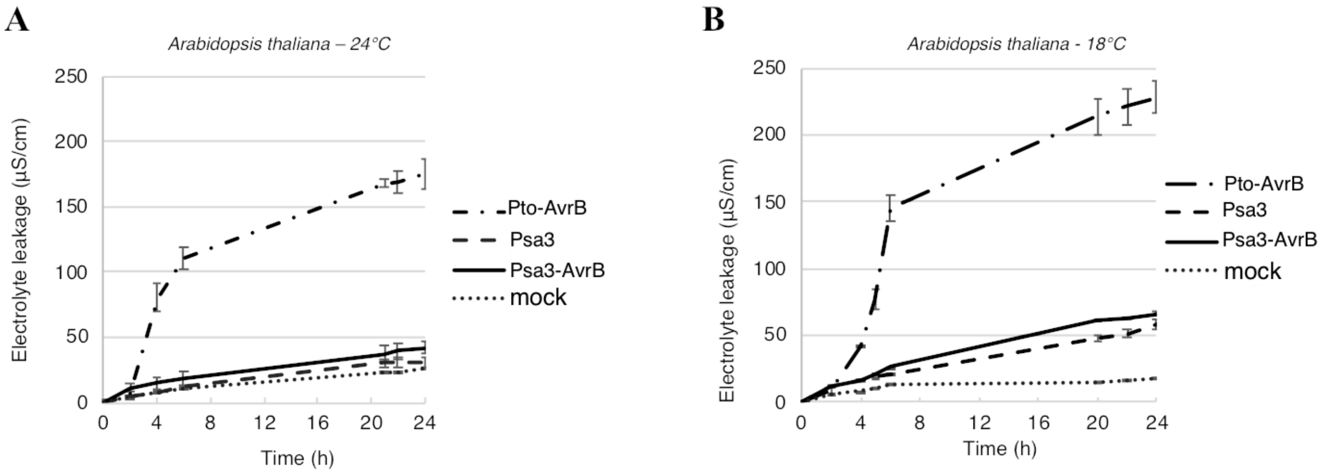

Supplementary Fig. S7. Time course of electrolyte leakage in *Arabidopsis thaliana* Col-0 leaf disks infiltrated with avirulent *P. syringae* pv. *tomato* (Pto) DC3000 or *P. syringae* pv. *actinidiae* biovar 3 (Psa3) at different temperatures.

The *A. thaliana* Col-0 leaf disks were infiltrated with wild-type Psa3, a Psa3 strain carrying the *AvrB* gene (Psa3-AvrB) or a Pto strain carrying the same gene (Pto-AvrB), and were incubated at 24 °C (A) or 18 °C (B). Leaf disks infiltrated with $MgCl_2$ were used as negative controls. Values correspond to the mean of three biological replicates, each including three technical replicates, ± standard error.

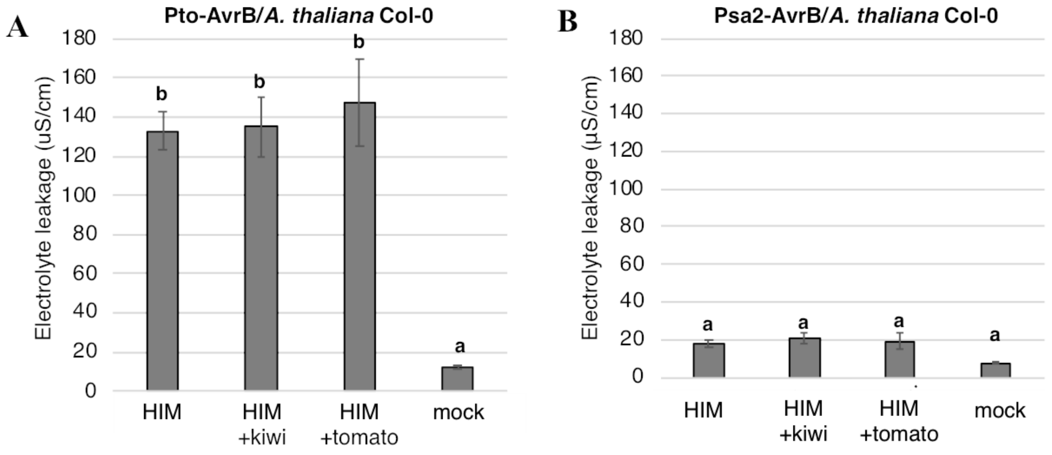

Supplementary Fig. S8. Electrolyte leakage in *Arabidopsis thaliana* Col-0 leaf disks 6 h post-infiltration with avirulent *P. syringae* pv. *tomato* (Pto) DC3000 or avirulent *P. syringae* pv. *actinidiae* biovar 2 (Psa2) pre-incubated in minimal medium (HIM) alone or in HIM supplemented with kiwifruit (+kiwi) or tomato (+tomato) extract.

Values correspond to the mean of three biological replicates, each including three technical replicates, ± standard error. Different letters indicate statistical differences according to an analysis of variance (ANOVA) test ($p < 0.05$).
